# Suspected cow’s milk allergy in everyday general practice: a retrospective cohort study on health care burden and guideline adherence

**DOI:** 10.1186/1756-0500-7-507

**Published:** 2014-08-09

**Authors:** Sharayke CTA van den Hoogen, Alma C van de Pol, Yolanda Meijer, Jaap Toet, Céline van Klei, Niek J de Wit

**Affiliations:** Julius Center for Health Sciences and Primary Care, University Medical Center Utrecht, Room Str 6.131 Postbus 85500, 3508 Utrecht, GA The Netherlands; Department of Paediatric Pulmonology and Allergology, University Medical Center Utrecht, Utrecht, 3508 AB The Netherlands; Department of Health Promotion & Epidemiology, Municipal Health Services Utrecht, Utrecht, 3521 AN The Netherlands; Preventive Child Health Care, Municipal Health Services Utrecht, Utrecht, 3521 AN The Netherlands

**Keywords:** Food hypersensitivity, Primary care, Prevalence, Diagnostic, Guideline adherence

## Abstract

**Background:**

Cow’s milk allergy (CMA) is the most common food allergy among infants. No data are available on the health care burden of suspected CMA in general practice. This study was conducted to evaluate the burden of suspected CMA in general practice (GP): (a) prevalence, (b) presenting symptoms, (c) diagnostic process, (d) guideline adherence, and (e) dietary measures.

**Methods:**

A retrospective cohort study was carried out in four Julius Healthcare Centers (JHCs). These JHCs form the core primary care academic network of the department of general practice of the University Medical Center of Utrecht. Electronic records of the first year of infants born May 2009 - April 2010 registered in the JHCs were screened for possible CMA suspicion. Preventive child healthcare (PCH) records were reviewed for additional information. Clinical presentation, diagnostic strategies and dietary measures were extracted.

**Results:**

Of 804 infants evaluated, 55 presented with symptoms fitting the suspicion of CMA (prevalence of 7%). Presenting complaints involved the skin (71%); the gastrointestinal tract (60%); the respiratory tract (13%) or other symptoms (36%) and 23 infants presented with symptoms of two or more organ systems. In 31 children (56%) a food challenge was performed (n = 28 open and n = 3 double-blind). Open challenge test results were difficult to interpret due to inadequate implementation or reporting. None had confirmed CMA after an adequate challenge test. Long term milk substitute formulas were prescribed in 39 (71%) infants.

**Conclusion:**

On a yearly basis seven percent of children visit their GP for suspected CMA. A positive CMA diagnosis was rarely established after adequate implementation and reporting of diagnostics, yet long term dietary measures were prescribed in >70% of patients. There is definitely need for improvement of diagnosing CMA in primary care.

## Background

Cow’s milk allergy (CMA) is the most common food allergy among infants with prevalences ranging from <1% (skin prick testing, blood tests and/or food challenges are used for diagnosis) to 3.5% (parent reported) [1, 2]. In primary care in the Netherlands the recommended diagnostic work-up for children with suspected CMA at the time of our study was an open food challenge (OFC), before starting long term diet. The consecutive steps of an OFC are described in detail in the current guideline "Food Hypersensitivity" issued by the Dutch College of General Practitioners (2010) [3]: elimination phase, provocation phase, and re-elimination of cow’s milk. When symptoms disappear after elimination, appear again after provocation, and subsequently disappear again after re-elimination a positive diagnosis of CMA is established. The preceding guideline on CMA (1995) was similar to the current one, yet the OFC procedure was described more generally [4]. In secondary care, a diagnosis is made by double-blind placebo controlled food challenge testing (DBPCFC) which is the gold standard in diagnosing a food allergy [5]. Last year, a new multidisciplinary guideline has been issued by the Dutch Association of Pediatrics, and insight in adherence to the former guideline may help identify room for improvement when implementing new recommendations [6].

Currently, the number of patients treated for CMA is presumably two to three times higher than justified [7]. An (unjustified) CMA diagnosis may have major impact both medically (somatisation, dietary deficiencies, growth delay etc.) [3, 4, 7] and economically (1500–2500 Euros per suspected patient in the first year after initial presentation [8, 9]). In the Dutch primary health care system management of suspected CMA is a shared responsibility by the GP and the preventive child healthcare (PCH). PCH is the Dutch national local health service whose role is routinely performing well-child visits throughout the first four years of life.

This paper tries to tackle a question raised in the NICE clinical guideline 116 (February 2011) [10]: What is the prevalence of suspected food allergy in children presenting to primary care? A suspected diagnosis is likely to be much more prevalent than (partially) confirmed CMA [2], as this is the nature of forming a diagnosis in primary care. Adequate work-up and follow-up policy for suspected CMA is important to make sure long term dietary measures are only taken for children that will benefit. We set out to determine the health care burden of infants suspected of CMA including prevalence, clinical presentation, diagnostic work-up, dietary measures and the national guideline adherence.

## Methods

### Design

Retrospective cohort study.

### Setting

The study was conducted in the four Julius Healthcare Centers (JHCs), primary care health centers with 35.000 patients and 23 GPs. JHCs form the core primary care academic network of the department of general practice of the University Medical Center of Utrecht. The patient population largely resembles that of the average Dutch population, although the under 18 population is somewhat larger: 30% <18 years old as compared to 20% for the wider Utrecht region [11]. Since other characteristics such as education level and ethnicity distributions are similar [11], we believe that the younger population in our area makes it even more suitable for primary care research projects regarding children. The primary and preventive health care system in the area under study is comparable to the rest of the Netherlands. Routine patient data are registered electronically since more than 10 years, using ICPC codes for diagnosis. During this study period the ICPC-1 codes were used instead of the recent published ICPC-2-E codes. GPs were trained in systematic data registration.

### Patients

The study population consisted of infants born between May 2009 and April 2010 registered in one of the four JHCs serving Leidsche Rijn. Leidsche Rijn is a rapidly growing new suburban area in the city of Utrecht in the Netherlands. In the surveyed period almost 70% of the residents of this district were of native origin. The large majority finished a secondary (25%) or academic (55%) education. Three quarters (75%) had paid work. Most resident had a high score on personal and social wellbeing [11].

### Definition

Suspected CMA was defined as: (a) recording of "a suspicion of CMA" in the healthcare record, and/or (b) implementation of CMA diagnostics and/or (c) implementation of CMA dietary measures (milk substitute formulas).

### Ethics

According to the Medical Ethical Research Council of our institution, there was no need for patient consent/ethical approval for this anonymous retrospective chart review.

### Data collection

Electronic records of GP consultations were retrieved to identify infants with suspected CMA in two steps. Since CMA (suspicion) is not an existing ICPC-1 code [12] we used a list of codes (symptoms of e.g. skin, respiratory tract, digestive tract; Table 1) to identify infants that had visited with complaints that could possibly fit a suspicion of CMA and subsequently reviewed their anonymous consult information to see whether CMA was indeed suspected (see definition above). Second, PCH records of infants with a potential suspicion of CMA were reviewed to confirm the suspicion and obtain additional information. The record-linkage of GP and PCH records was not performed by the investigator. For infants suspected of CMA the following variables were abstracted from their GP and PCH records: sex, age at onset of the suspicion, presenting complaints (skin, gastro-intestinal, respiratory, circulatory or other), diagnostic tests performed, and dietary measures. Diagnostics were defined as: a DBPCFC, an OFC and a radioallergosorbent blood test (RAST). According to the current guideline "Food Hypersensitivity" an OFC is correctly conducted when the following phases have been conducted: (a) symptoms are assessed at start of elimination and at start and end of the provocation phase (b) the test was continued for at least one week in case of mild symptoms during provocation (red stains around the mouth, crying or mild eczema) (c) the test was interpreted as positive only when original symptoms returned after provocation and again disappeared after re-elimination. For each OFC the recorded procedures for each phase were assessed to evaluate whether guideline recommendations had been followed [3]. Finally, dietary measures were recorded (types of milk formulas prescribed).

**Table 1:** **ICPC-1 codes used to identify infants who had visited with symptoms potentially suggestive of CMA**

A12 Allergy	R02 Dyspnoea
A14 Colics in infants	R03 Wheezing
A15 Excessive crying infant	R07 Sneeze, rhinitis, nasal discharge
A16 Irritable/hyperactive infant	R29 Other symptoms respiratory tract
A17 General symptoms/complaints infant	R96.01 Airway hyperreactivity
A29 Other general symptoms	R97 Allergic rhinitis
D10 Vomiting	S06 Local redness/erythema skin
D11 Diarrhea	S07 Generalized redness/erythema skin
D12 Constipation	S21 Other symptoms/complaints aspect skin
D16 Rectal bleeding	S29 Other symptoms/complaints skin/subcutis
D18 Altered defecation	S87 Constitutional eczema
D20 Complaints mouth	S98 Urticaria
D29 Other symptoms/complaints gastrointestinal tract	S99 Other diseases skin/subcutis
F71 Allergic conjunctivitis	T04 Nutritional problems infant

## Results

### Population and prevalence

**Figure 1:**
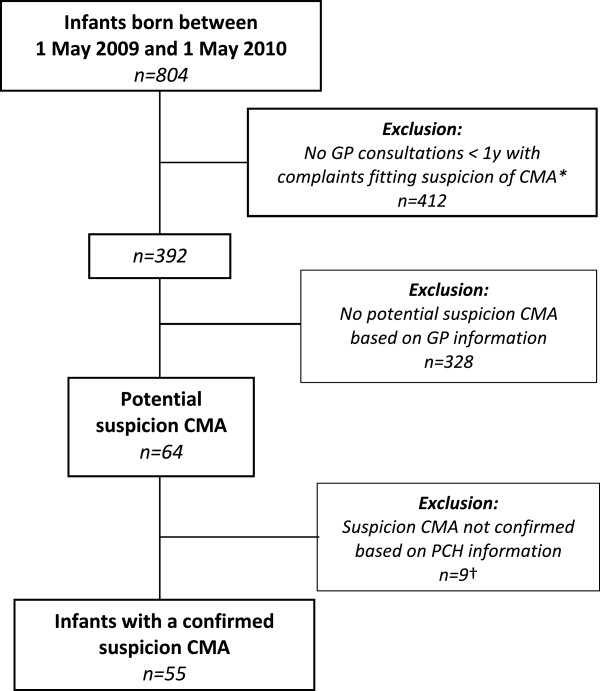
**Flow-chart: inclusion of infants.** Legend. *n =* number of infants. GP: general practitioner; y: year; CMA: cow’s milk allergy; PCH: preventive child health care. * For definition of a suspicion of CMA see Methods section. † Of which three suspicions not confirmed due to the lack of PCH records.

In total 804 infants born from May 2009 through April 2010 were registered in the four JHCs in Leidsche Rijn of which 392 infants (49%) visited their GP with symptoms that could possibly fit a suspicion of CMA (Table 1). After reviewing the GP’s notes of these visits 64 infants were identified with a potential suspicion, and their PCH records were screened for additional data. Finally, 55 infants were confirmed to have a suspicion of CMA, i.e. the prevalence of suspected CMA was found to be 7% (Figure 1).

### Clinical presentation of suspected of CMA

The median age of the 55 infants suspected of CMA was 14 weeks and 34 (62%) were male. At time of presenting with a suspicion of CMA 39 of the infants had complaints of the skin (56% eczema, 15% urticaria or rash); 33 of the gastrointestinal tract (22% vomiting, 24% colics, 7% diarrhea, none had constipation, 7% other: rectal bleeding or feeding problems); seven of the respiratory tract (6% respiratory infections, 7% stridor, none had rhinitis or cough). None of the infants showed circulatory tract symptoms (tachycardia, hypotension, collapse). Twenty infants had other type of symptoms (34% excessive crying and agitation, 2% fever). In 23 (42%) infants, symptoms of two or more organ systems were present at the time of presentation.

### Diagnostic process

#### Type of testing performed

In 40 of the 55 CMA suspected infants additional diagnostics were undertaken: a DBPCFC in n = 3 (all by pediatrician), an OFC in n = 28 (50% GP, 39% PCH professional, 4% pediatrician, 7% unknown) and a RAST in n = 15 (47% GP, 47% pediatrician, 6% unknown).

#### Elimination/provocation

**Figure 2:**
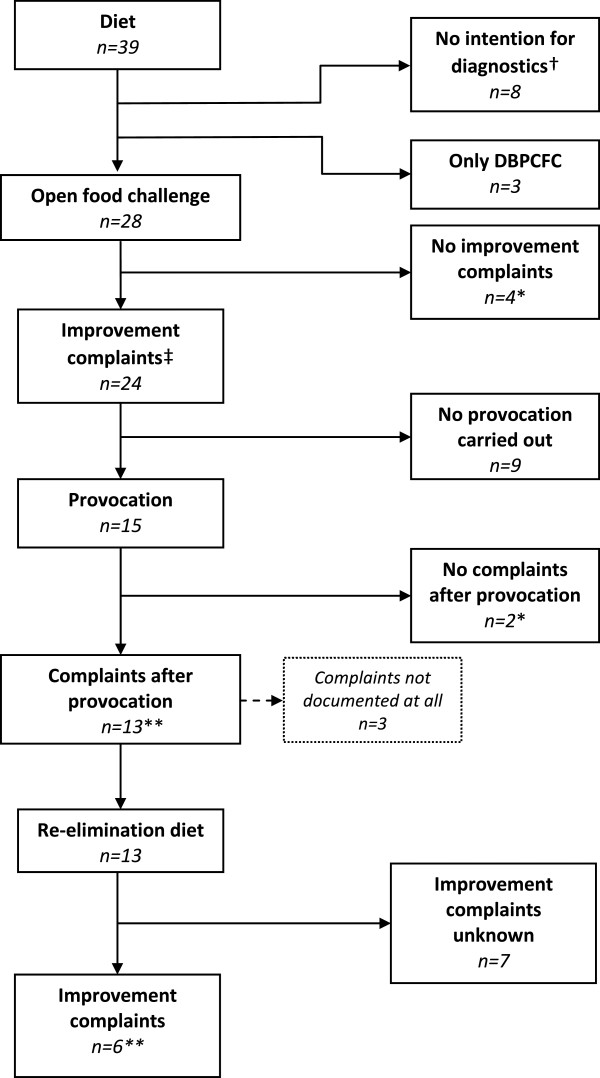
**Flow chart: Infants which followed a diet and/or open food challenge.** Legend. *n =* number of infants; DBPCFC = double-blind placebo controlled food challenge. † Not documented as starting diet with the intention of elimination and/or provocation or with the intention of assessment of complaints during diet. * Carried out and entirely documented following valid guideline recommendations [3, 4]. ‡ Eligible for provocation according the guideline [3, 4]. ** Not documented as carried out according to the guideline [3, 4].

Of the 28 OFCs performed n = 6 were negative. A total of n = 22 tests were inconclusive when checked for adherence with the current guideline [3], or adherence was unclear due to inadequate reporting. Of the 22 inconclusive results six were interpreted as positive by the clinician involved. Results of the separate phases of the OFC tests are set out in Figure 2. In 8 (21%) of the 39 infants who started with an elimination diet, it was not reported this was done in order to perform an OFC. Reasons for OFCs not being in adherence with guideline recommendations were (a) the suspicion of CMA not being substantial enough to warrant OFC [3], (b) not all test phases were (completely) performed, and (c) appearance or disappearance of complaints in the several phases of OFC was not documented. In summary, none of the infants had a positive DBPCFC or OFC entirely reported as performed according the guideline (Figure 2) [3].

### Dietary measures

A milk substitute formula was prescribed in 39 infants (3% whey hydrolysates milk formulas, 72% caseïne hydrolysates milk formulas, 3% aminoacid based milk formulas and 21% breast milk with an elimination diet for the mother). A third of the infants (33%) had used more than one type of milk substitute formulas. Duration of used dietary measures was not clear due to insufficient detailed information in both records.

## Discussion

### Summary

The prevalence of 7% for a suspicion of CMA found in the present study is 2–7 times higher compared with prevalence figures from previous studies assessing the prevalence of "diagnosed CMA" (ranging from <1% to 3.5%) [1, 2]. We showed that a suspicion of CMA is a common problem in general practice, and that there is room for improvement of implementation and reporting of CMA diagnostics (none of the infants had a confirmed CMA diagnosis after a challenge test performed and reported according to Dutch national guideline recommendations).

### Guideline adherence

CMA OFC test results were often difficult to interpret because the indication, implementation and reporting of diagnostics did not follow the current Dutch national guideline recommendations [3]. Therefore, the prevalence and health care burden of true (i.e. adequately confirmed) CMA in general practice was impossible to establish in this study. A possible explanation for common non-adherence to the definition of CMA suspicion could be parents who might be demanding towards their GP, insisting on additional diagnostic testing for CMA. However, in just a few cases (7%) the suspicion of CMA was brought up solely by parents. In nearly all cases (82%) the GP recorded that he or she at least shared the suspicion and acted accordingly.

### Limitations

By using a selection of ICPC-1 codes that could possibly prompt a suspicion of CMA (Table 1) it is not inconceivable that a small number of relevant GP visits may have been missed. Unfortunately, CMA symptom ICPC code screening could only be performed for GP electronic records, and not for PCH records since a digital coding system is not available for their records. It is, however, unlikely that suspected cases were missed, since children with a possible CMA presenting to their PCH professional are likely to present also to the GP, and if not the PCH professionals by protocol will inform the GP in writing of any CMA diagnostics being performed, which will then be coded by the GP in the electronic system. Besides we could not assess the total amount of dietary formula prescribed. We could assess the percentage of children using prescribed milk substitute formulas (71% of suspected children), though the duration of such formula use we could not evaluate resulting the lack of information in the records. Finally, our study was performed in four healthcare centers (23 GPs) in one central region of the Netherlands, possibly restricting generalizability of results.

### Implications

Prevalence data can be used for further development of health care policy and intervention studies. Our results show that a suspicion of CMA is common in general practice, and that diagnosing and reporting of CMA work-up needs to be improved. The recently issued multidisciplinary guideline for diagnosing CMA in the Netherlands can contribute when completely implemented [6]. Implementation in PCH is currently in progress [13]. Remaining questions to be addressed include possible explanations for non-adherence in the diagnosis of CMA, its consequences and what needs to be further done to improve these results.

## Conclusion

Suspected CMA is a common reason for consultation in primary care in the four Utrecht healthcare centers included in our study, with a prevalence of 7%. Current management, in particular the diagnostic part, is not according to professional guidelines and dietary measures are prescribed often for a long time but not evaluated appropriately.
